# National belonging and psychological strain among Finnish migrant populations

**DOI:** 10.1093/eurpub/ckac131.513

**Published:** 2022-10-25

**Authors:** A Seppänen, H Kuusio, L Mäkipää, E Lilja, S Rask, A Castaneda

**Affiliations:** Equality, Public Health and Welfare, Finnish Institute for Health and Welfare, Helsinki, Finland

## Abstract

**Background:**

National identities are socially constructed and imaginary groups with real-life consequences. Migrants are in a heightened risk to be treated as ‘others’ who do not belong to society. It remains unclear, to what extent national belonging is experienced among Finnish migrants and what consequences on health this has. We study migrants’ sense of belonging to Finns, how it’s manifested in sociodemographic groups and whether it’s associated with psychological strain.

**Methods:**

We used nationally representative data from the cross-sectional Survey on Well-Being among Foreign-Born Population (FinMonik, n = 6836). National belonging was assessed by the item “Finns” in question “which of the following areas or groups you feel you belong to?”. Response options fully and quite a lot were coded to indicate sense of belonging. Logistic regression was used to test the association between belonging, sociodemographic factors and psychological strain (MHI-5). Weights were used to correct the sample.

**Results:**

51% reported sense of belonging to Finns. 46% of those aged 30-44 reported sense of belonging to Finns, whereas the youngest and oldest age groups yielded highest prevalences (18-29=53% and 45-64=58%, p<.001). Married persons reported sense of belonging to Finns more than those who weren't (55% vs. 48%, p<.01). Country group accounted for the variation in Finnish identification with a p-value of less than 0.001. Only 27 percent of those born in East Asia reported sense of belonging to Finns, whereas almost 60% of those born in Middle East and North Africa sensed belonging to Finns. Those with sense of belonging to Finns were twice as likely to report lack of psychological strain than those with no sense of belonging to Finns (p<.001).

**Conclusions:**

Achieving national belonging to the receiving society seems to be more difficult or non-appealing for some migrant populations than others. Lack of national belonging poses risk of deterioration of mental health.

**Key messages:**

• The socially constructed boundaries of national belonging can be exclusionary and have negative consequences for the health of migrant populations.

• Experiencing a sense of national belonging to the country of residence has positive associations with mental wellbeing.

